# Sex-specific spatial use of the winter foraging areas by Magellanic penguins and assessment of potential conflicts with fisheries during winter dispersal

**DOI:** 10.1371/journal.pone.0256339

**Published:** 2021-08-20

**Authors:** Samanta Dodino, Nicolás A. Lois, Luciana Riccialdelli, Michael J. Polito, Klemens Pütz, Andrea Raya Rey

**Affiliations:** 1 Ecología y Conservación de Vida Silvestre, Centro Austral de Investigaciones Científicas, Consejo Nacional de Investigaciones Científicas y Técnicas, Ushuaia, Tierra del Fuego, Argentina; 2 Instituto de Ecología, Genética y Evolución de Buenos Aires, Consejo Nacional de Investigaciones Científicas y Técnicas, Buenos Aires, Argentina; 3 Departamento de Ecología, Genética y Evolución, Facultad de Ciencias Exactas y Naturales, Universidad de Buenos Aires, Buenos Aires, Argentina; 4 Department of Oceanography and Coastal Sciences, Louisiana State University, Baton Rouge, Louisiana, United States of America; 5 Antarctic Research Trust, Bremervörde, Germany; 6 Instituto de Ciencias Polares, Ambiente y Recursos Naturales, Universidad Nacional de Tierra del Fuego, Ushuaia, Argentina; 7 Wildlife Conservation Society, Buenos Aires, Argentina; MARE – Marine and Environmental Sciences Centre, PORTUGAL

## Abstract

Magellanic penguins (*Spheniscus magellanicus*) disperse widely during winter and are a major consumer of marine resources over the Patagonian Shelf. Magellanic penguins were equipped with geolocators at Martillo Island in late February- early March 2017 and recaptured at the beginning of the next breeding season to recover the devices and to collect blood samples for stable carbon (δ^13^C) and nitrogen (δ^15^N) isotope analysis. We evaluated their whole winter dispersal and their trophic niche by sex during the last month of the winter dispersal. Also, we evaluated their spatial overlap with bottom trawl and shrimp fisheries using data from satellite fisheries monitoring. Penguins dispersed northwards up to 42°S and showed latitudinal spatial segregation between sexes during May to August (females were located further north than males). In contrast, during the last month of the winter dispersal females were located more southerly and showed lower trophic position than males. Also, females did not dive as deep as males during winter. We found high overlap between both fisheries and penguin’s spatial use in regions with documented interaction. However, no sex-specific statistical differences with fisheries overlap were found. Our results highlight the importance of understanding the spatial domains of each sex and assessment of their potential conflicts with bottom trawl fishery and shrimp fishery during the winter period.

## Introduction

A large number of species synchronize their movements with the availability of their main food sources found in geographically distant areas [1–3] and change their niche throughout the year, especially in spatial dimensions. During non-reproductive periods, many seabirds undertake dispersal movements at sea to feed without returning to land [4]. Studies with geolocators have reported differences between sexes in phenology (start or end dates), destination areas and latitudinal ranges used during this period [5, 6]. In addition, studies based on stable isotopes have identified sexual niche partitioning during this period as well [7, 8].

The non-reproductive period represents the time of highest risk for seabirds [9] and is crucial for the breeding success in the next season as it can influence the body condition of breeders at the time of arrival in the colony [10]. Characterizing dispersal movements and trophic ecology during that period may contribute to the conservation of seabird species, as it can provide information on foraging areas [11], their role in the food web, and overlap with human activities [12, 13]. Moreover, the assessment of human activities in the ocean and understanding their interaction with seabirds is an important step in the development of adequate conservation plans for the creation and management of Marine Protected Areas (MPA) and/or the implementation of mitigation measures. In this sense, seabird-fisheries interactions are currently a major issue in marine conservation management [14].

Magellanic penguins (*Spheniscus magellanicus*) disperse widely during winter and are a major consumer of marine resources [15] over the Patagonian Shelf (PS), which extends off the coast of Argentina from its southern tip to 40°S to the slope at 200 m isobath [16]. Winter dispersal sets off by the end of March/beginning of April and penguins stay approximately six months at sea before returning to the colony for the next breeding season (mid to late September). Three studies have focused on this species winter dispersal in the PS so far. Two studies in Northern Patagonia have successfully recorded Magellanic penguin throughout their whole winter dispersal and reported differences in the spatial use between sexes [17, 18] but no differences in trophic position based on blood tissue δ^15^N values [18]. While in southern Patagonia, winter dispersal was recorded from March to June and showed no differences in spatial use by sex during those months [19].

Seabirds-fisheries interactions are a result of the overlap between the foraging areas used by seabirds and the areas used by the fisheries. Fisheries by-catch mortality has been documented for Magellanic penguins all along its distributional range, especially in areas of high fishing effort during the autumn and winter periods off the coast of Argentina (e.g., [20–22]. Also, a female-biased mortality was found in carcasses of Magellanic penguins’ overwintering grounds in southern Brazil among individuals died by starvation [23], and a female-biased mortality in by-catch of gillnet fisheries [24]. In northern Patagonia, a study reported that the non-breeding-season survival of females was lower than in males, thereby influencing population growth rate [25]. Therefore, sex plays a significant role in the movement ecology of this species and its conservation.

In addition, inshore foraging flightless seabirds, such as penguins, depend on the presence of abundant and predictable food resources, e.g. crustaceans and fish stocks [26]. Previous studies reported overlaps between Magellanic penguins’ diet and fishery activities on the Patagonian Shelf [27, 28]. The prey resources for which Magellanic penguins overlap with fisheries catch are: anchovy (*Engraulis anchoita*), common hake (*Merluccius hubbsi*), Patagonian toothfish (*Dissostichus eleginoides*), southern blue whiting (*Micromesistius australis*), squid (*Illex argentinus*, *Doryteuthis gahi*), shrimps (*Pleoticus muelleri*, *Peisos petrunkevitch*), and potentially Fuegian sprat (*Sprattus fuegensis*) and squat lobster (*Munida gregaria*) [29]. Competition between birds and fisheries may not occur if the resource is abundant (i.e. not limited, [30]). However, these target species of commercial and artisanal fisheries have been overexploited for decades in the Argentine Sea [31, 32], so direct and indirect competition between seabirds and fisheries is likely.

In this context, our goals were to (1) analyze the sex-specific spatial use of Magellanic penguins from Martillo Island during the whole winter dispersal, (2) analyze their trophic niche by sex during the last month of the winter dispersal, and (3) assess the potential spatial overlap with fisheries over the PS. To this aim we leverage geolocator sensors (GLS) data together with stable isotopes analysis (δ^13^C and δ^15^N) and satellite fisheries monitoring. We hypothesize that Magellanic penguins exhibit sexual segregation in foraging behavior. We thus expect males and females to use different areas and portray differences in their isotopic niches. Fishing activities in the area are spatially and temporarily clustered, which drives us to expect a sex-biased overlap with fishing activities during this species’ winter dispersal through the PS related to sex-specific spatial-niche partitioning.

## Methods

### Study area, tracking technique and sample collection

We conducted field work at the Magellanic penguin colony on Martillo Island (54° 54 ’S, 67° 23’W). This island is located in the eastern section of the Beagle Channel (Tierra del Fuego, Argentina) and holds ca. 4900 active nests (A. Raya Rey unpubl. data).

We tracked penguins’ non-breeding movements using light-based geolocation techniques via LAT2900 geolocators (global location sensing loggers, GLS, Lotek Wireless). We attached the devices to the penguin’s tarsus between late February and early March 2017 (13 adult females and 13 adult males) using cable ties. In order to increase the likelihood of successful recovery the following year, we chose breeding penguins that were tagged with transponders (Raya Rey et al. 2007) in previous seasons, and also identified their nests with wooden stakes. Sex determination was based on differences in beak widths and lengths [33]. We recovered the devices at the onset of the following breeding season when penguins returned to the colony (September-October 2017) and collected 1 ml whole-blood samples from the tarsal vein (the maximum time between penguins’ arrival and device recovery was 3 days). We preserved blood samples in vials with 70% ethanol until further processing in laboratory [34].

This study was evaluated and approved by the Wildlife Direction, Environmental Secretary, Tierra del Fuego Government taking into account animal research ethic perspective with Argentinian Government permission: Resol. SUB. P.A.y S. N° 014/2017, "Trophic and genetic ecology of seabirds’ assemblage from the Beagle Channel and Staten Island: spatial and temporal variation".

All statistical analyses were performed in R software ver. 3.6.3 [35]. We considered a significance to occur at p< 0.05 and all means are presented ± standard deviation (SD).

### Track analysis

We downloaded the information collected by the devices using the software provided by Lotek SA (Tag Talk Software). LAT 2900 geolocators record daily estimates of absolute times of sunrise and sunset. The daily locations are estimated based on a template-fit algorithm [36] and latitudes are derived from day lengths and longitudes from the relative timing of recorded midday or midnight [37]. In order to improve the accuracy of the daily locations, we re-estimated them using the R package ProbGLS [38]. This package uses an iterative forward step selection process and does not require the assumption or calibration of a constant solar angle throughout the year [38]. Also, this method allows estimating positions even during the equinox periods when light-based geolocation estimates are difficult to obtain due to minimal variation in day length around the world. The algorithm calculates a cloud of possible locations by applying a range of solar angles using a number of particles at each time step (we selected 2000 particles following [38] and weighing each particle according to animal behavior (Magellanic penguin’s travelling speed; [10, 39]). In addition, this analysis takes into account background environmental characteristics such as a sea surface temperature (SST) and a land mask (0.25° × 0.25° NOAA OI SST V2 High Resolution Dataset). This allows to match geolocators temperature records (we used the daily lowest surface water temperatures following [17]) with satellite SST data and land avoidance for marine species [40, 41]. Finally, the algorithm iterates a preset number of times (in this study, n = 200) to construct several probable movement paths, and computing the most likely movement path as the geographic median for each location cloud (for more details see [38]). These estimated locations allow for realistic speed and distance metrics, reducing the effects of the inherent lower accuracy of using geolocation. This approach reduces errors in latitude to medians of 185 km and 145 km for solstice and equinox periods, respectively [38].

In addition to the daily locations, geolocators were set to record the daily maximum diving depth and also the wet–dry state (0: wet, 1: dry) at 30 minutes intervals throughout the recording period. The wet/dry state allowed estimating the beginning (always wet) and the end (mostly dry) of the winter dispersal.

### Data track analysis

We estimated the latitudinal range for each penguin calculated as the distance from the northernmost and southernmost latitudinal points recorded in a straight line with the function ‘distm’ from the package ‘geosphere’ [35]. We evaluated differences in the latitudinal range between sexes using generalized least square models (GLS) (the groups are non-equilibrated and the variance is not homogenous) with varIdent variance function (nlme package; [42]). The response variable was the latitudinal range and the explanatory variable was sex.

Moreover, we evaluated differences between sexes in trip duration (the time that penguins spent at sea during winter dispersion) using GLS with varIdent variance function where the response variable was the trip duration and the explanatory variable was sex. On the other hand, we examined sexual differences in the daily maximum diving depths along all the winter dispersal using generalized linear mixed models (GLMM) with a Gaussian distribution, varIdent variance function and penguin identity as a random factor (nlme package; [42]).

### Spatial analysis and fisheries overlap

The Ministry of Agriculture, Livestock and Fisheries of Argentina (MAGyP, Spanish acronym) provided information on fishing hours according to satellite monitoring of vessels operating from April to September 2017 between 38°S and 60°S and from the coast up to 200 miles. The Argentinean Vessel Monitoring System provides the GPS position of each vessel every 60 min. Fishing hours where estimated by taking into account only boats with speeds compatible with fishing (2−5 knots) (0.5° × 0.5° resolution, [43]), hereafter fishing effort. Positions obtained only during daylight were used, as fishing takes place only during the day. Then, the data was filtered by fisheries which could affect penguins. Forage-divers such us penguins take advantage of discards using the pursuit diving strategy, obtaining discards directly from or falling off the net during haul-back, which increases their vulnerability to become entangled [44, 45]. Taking this into account together with interactions previously reported in this area, we selected bottom trawl fisheries (which includes fisheries targeting predominantly finfish species) and shrimp fishery (Magellanic penguins might suffer by-catch by both fisheries and their diet overlaps with fisheries’ target species, [27, 45]). We conducted separate analysis for each fishery, taking into account that these fisheries showed different spatial distribution in the Patagonian shelf (see S3 Fig).

Penguin positions and fisheries data were analyzed in Python environment ver. 3.7.8 (packages cartopy 0.18.0, cmocean 2, descartes 1.10., earthpy 0.9.2, geopandas 0.6.1, matplotlib 3.3.1, numpy 1.18.1, pandas 0.25.3, scipy 1.3.2, xarray 0.14.1) through the creation of density maps based on a 2° by 2° grid, which was generated taking into account the approximate error of the daily locations (see Phyton script in S1 Code). A study using a similar approach, spatial scale and combining Northern fulmars geolocators data with fishing effort data in the North Atlantic has been recently published [46]. We counted location fixes within each cell and, due to the unbalance in location number between sexes (data location of 5 females and 4 males were recorded successfully), we normalized the count to the maximum count for each sex and month. Also, we plotted a density distribution map for the last months of the winter dispersal (end-August/ beginning-September) separated by sexes in order to evaluate a possible correlation between the spatial distribution and the information provided by the stable isotope composition of blood samples. Taking into account stable isotope turnover in penguin blood (~30 days, [47]), the last month was defined as 30 days prior to the date of blood sample collection.

We re-gridded fishing effort to the 2° by 2° grid used for penguin location density estimation. Due to the uneven distribution of monthly fishing effort and the existence of pixels with extreme values, we associated color categories based on a fishing score from 0 to 5 that coincide with the ranges of fishing effort values used by MAGyP (ranges: 1: 0–60, 2: 60–200, 3: 200–800, 4: 800–6000, 5: 6000–10000) [43].

We calculated a binomial variable from the pixels in which penguins and fisheries were present (overlap = 1) and the pixels in which penguins were present but there was no fishing effort (overlap = 0). Also, to compute the potential degree of interaction between penguins and fisheries, we calculated the interaction for each sex (range: 0–5) as:

interaction=penguindensity*fishingeffortscore

We evaluated differences between sexes in fisheries overlap using the generalized lineal model with Binomial distribution. The response variable was the overlap (overlap = 1, no overlap = 0) and the explanatory variable was the sex. Also, we examined differences between sexes in interaction with fisheries using the generalized lineal model with Gaussian distribution. The response variable was the interaction, previously calculated, and the explanatory variable was the sex. In all models, we conducted separated analysis for bottom trawl fisheries and shrimp fishery.

### Stable isotope analysis

We dried the blood samples in an oven at 60°C for *ca*. 24h to remove ethanol and then samples were lyophilized and homogenized. Homogenized blood samples were weighed (0.6 mg ± 0.1 mg) into tin cups, flash-combusted (Costech ECS 4010 elemental analyzers) and analyzed for carbon (δ^13^C) and nitrogen (δ^15^N) stable isotope values via an interfaced Delta XP continuous-flow stable isotope ratio mass spectrometer at Louisiana State University. USGS 40 and USGS 41 glutamic acid reference materials were used to normalize sample values. Sample precision based on repeated sample and reference material was 0.1‰ for both δ^13^C and δ^15^N. Stable isotope values are expressed in δ notation in per mil units (‰), according to the following equation:

$$\delta X=[(R_{sample}/R_{standard})-1]$$

where X represents either ^13^C and ^15^N and R the ratio between ^15^N/^13^N or ^13^C/^12^C. R_standard_ for δ^15^N was based on atmospheric N_2_ while for δ^13^C was based on Pee Dee Belemnite (PDB).

### Isotopic niche analysis

In order to assess differences between sexes we examined δ^13^C and δ^15^N values from blood samples using separate generalized least square models (GLS) (the groups are non-equilibrated and the variance is not homogenous) with varIdent variance function (nlme package ver. 3.1–151; [42]). The response variable was the isotope value, and the explanatory variable was sex. We used one model for δ^13^C and another for δ^15^N.

Also, we estimated the isotopic niche width of each sex using standardized ellipse areas (SEA) corrected for sample size (SEA_C_) and Bayesian standard ellipse area (SEA_B_) based on δ^13^C and δ^15^N values. These metrics avoid the tendency for underestimation of isotopic niche width at small sample sizes and provide credibility intervals to aid in quantifying uncertainty [48]. We calculated SEA_C_ and SEA_B_ using the Stable Isotope Bayesian Ellipses in R (SIBER, [48]) package which allowed us to calculate the posterior probability that one group has a smaller isotopic niche width than the other group. We used SEA_C_ for graphical representations for calculating the percentage of isotopic area overlap between sexes.

### Trophic position estimates

We estimated the trophic position (TP) of each sex implementing a Bayesian approach using the full models of the *tRophicPosition* package in R [49]. The geolocators analysis for the last month before penguins returns to the colony allowed improving the TP estimation considering one baseline for males, and two for females (see Results, “Spatial analysis and fisheries interaction”). For males we selected: 1) Northern Patagonia baseline: mean δ^15^N values (11.6 ± 0.1 ‰) and mean δ^13^C values (-18.7 ± 0.5‰) of *Aulacomya atra* (sampled from the northern sector of Patagonia 40° S to 44° S; [50]). For females we selected: 2) Southern Patagonia baseline: mean δ^15^N values (12.7 ± 0.5 ‰) and mean δ^13^C values (-15.8 ± 0.5‰) of *Aulacomya atra* (sampled from the southern sector of Patagonia 48° S to 52° S, [50]) and 3) Bahía Franklin baseline: mean δ^15^N values (10.8 ± 0.5 ‰) and mean δ^13^C values (-14.7 ± 0.3‰) of *Mytilus chilensis* (sampled from Bahía Franklin, Isla de los Estados 54° 47’ S, in front of Atlantic cost of Tierra del Fuego, [51]). We assumed all bivalves were completely herbivorous and occupied a TP of 2. Since *Aulacomya atra* samples were collected in 2009, we applied a correction factor of -0.022 ‰ year-1 to all carbon stable isotope sample values to account for the Suess effect [52, 53]. We also assume that the δ^15^N values of the bivalves did not change over the years. We used the trophic discrimination factor (TDF) of 2.8 ± 0.2 ‰ for δ^15^N values and 0.9 ± 0.1 for δ^13^C values estimated for blood samples in Magellanic penguins [54]. We evaluated differences between sexes using the *compareTwoDistributions* function [49].

## Results

We recaptured 24 out of 26 adults (92% recapture rate, 13 females and 11 males) and collected blood samples from all of them. However, only 20 penguins were still equipped with devices and only 9 datasets could be recovered successfully due to software issues (S1 Dataset).

### Track analysis

**Fig 1:**
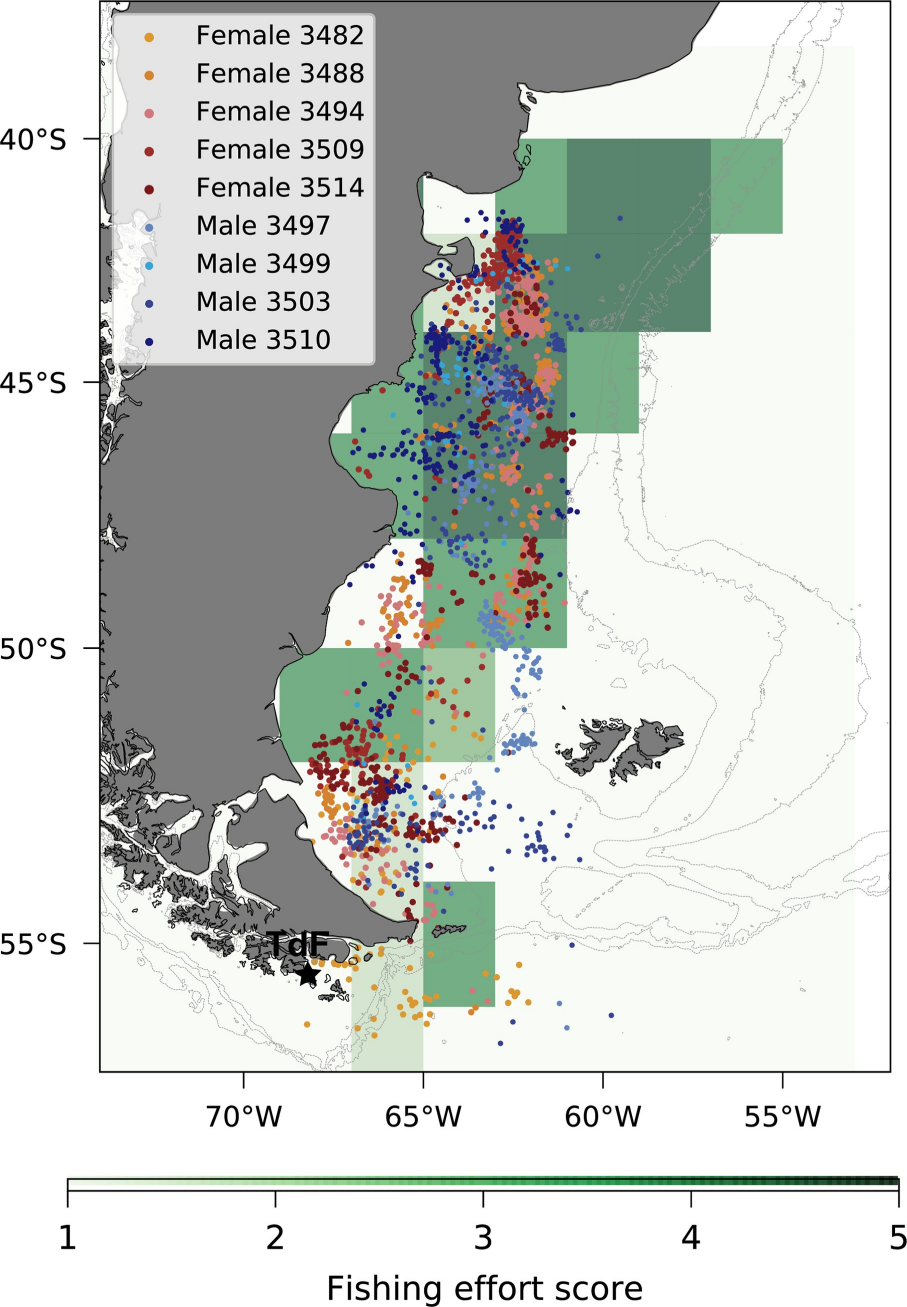
Movements of female and male Magellanic penguins overlaid to bottom trawl and shrimp fisheries density (2° latitude x 2° longitude) during winter dispersal (April- September 2017). The star indicates the location of the study site (Martillo Island, Tierra del Fuego Province, TdF) situated in the southernmost range of Magellanic penguin breeding distribution in Argentina. Colored color bar is used for females and a blue color bar for males. Score: 1: 0–60, 2: 60–200, 3: 200–800, 4: 800–6000, 5: 6000–10000. Light gray isobaths were obtained from [55, 56]. Fisheries data were obtained from MAGyP [43].

We could record the winter dispersal of 5 female and 4 male Magellanic penguins between April and September 2017. Latitudinally, penguins dispersed in an area ranging northwards to 42°S at the Península Valdés in Chubut Province and southwards to 56°S past Tierra del Fuego Province (Fig 1). However, one female did not migrate further north than 50°S (female 3482, S1 Fig). Females left the colony between 16 March and 3 April and returned between 18 September and 2 October, while males left the colony between 29 and 31 March and returned between 14 and 23 September (Table 1).

**Table 1:** Summary of details from Magellanic penguins equipped with geolocators at Martillo Island: Dates of departure and arrivals to the colony, and trip durations (days). Also, for each penguin, latitudinal range (km) during the winter period is shown, estimated as the distance between the northernmost and southernmost location recorded.

N°GLS	Sex	Departure date	Arrival date	Trip duration (days)	Latitudinal range (km)
3482	Female	16/03/2017	18/09/2017	187	578.3
3488	Female	16/03/2017	25/09/2017	194	1514.9
3494	Female	22/03/2017	02/10/2017	195	1486.5
3509	Female	03/04/2017	21/09/2017	172	1407.9
3514	Female	29/03/2017	21/09/2017	177	1385.4
3497	Male	31/03/2017	20/09/2017	174	1327.7
3499	Male	29/03/2017	16/09/2017	172	1588.3
3503	Male	29/03/2017	23/09/2017	179	1672.5
3510	Male	31/03/2017	14/09/2017	168	1303.5

We did not find sex-related differences in trip duration (*F*_*1*,*7*_ = 5.20, p = 0.06, females: 185 ± 10.22 days, males: 173.25 ± 14.57 days, Table 1) or latitudinal range (*F*_*1*,*7*_ = 0.35, p = 0.36, females: 1274.6 ± 392.9 km, males: 1473 ± 185.2 km, Table 1). We did find differences in the daily maximum depth with males reaching deeper waters than females: 57.1 ± 18.2 m and 44.3 ± 12.1 m respectively (GLMM, estimate = 44.27, SE = 12.11, t = 3.66, p = 0.04, CI: [30.13–55.81]).

### Spatial analysis and fisheries overlap

**Fig 2:**
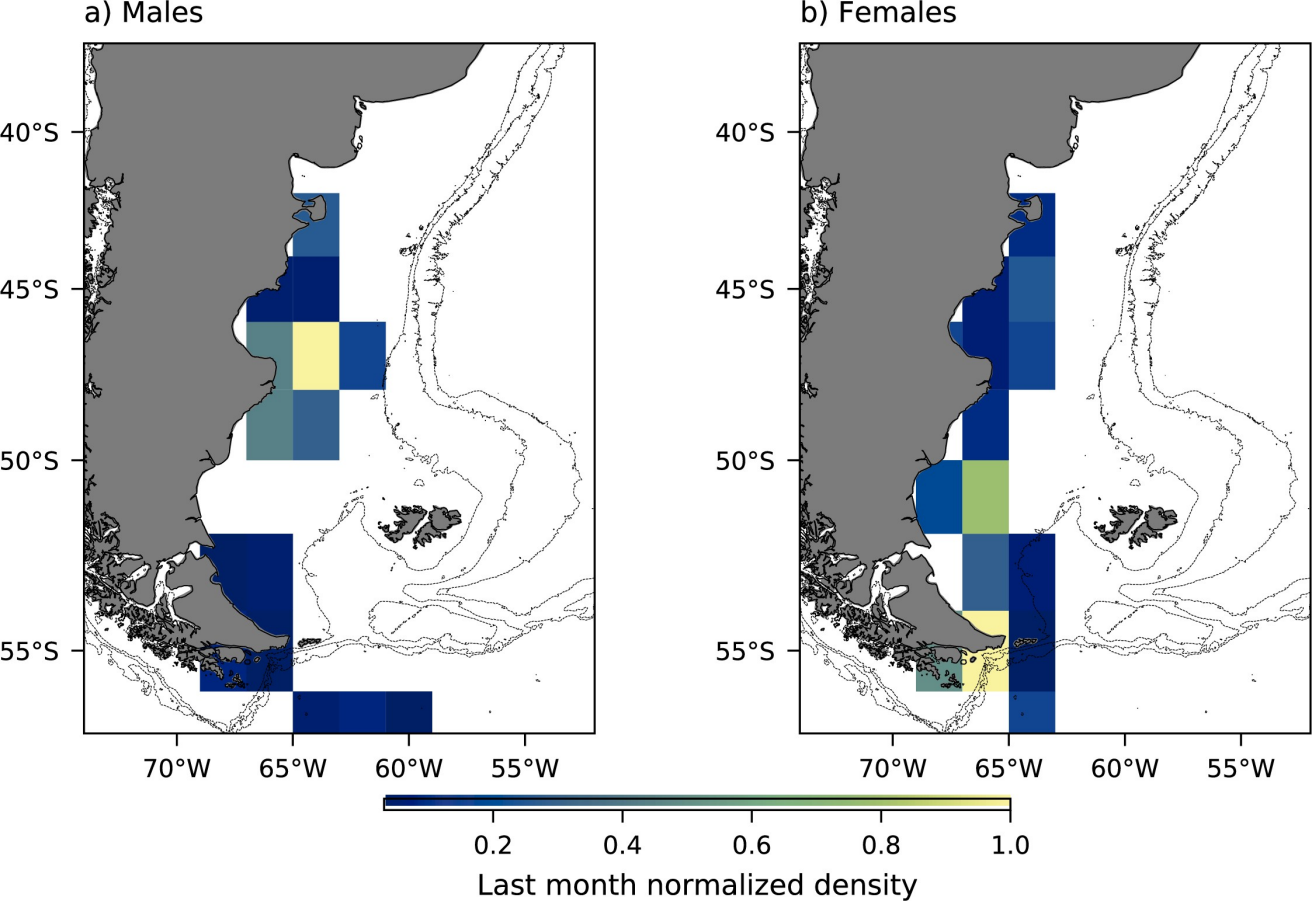
Density plot of female and male Magellanic penguins from Martillo Island during the last month of their winter dispersal (end August- beginning September 2017, grid square: 2° latitude x 2° longitude). Light gray isobaths were obtained from [55, 56].

During April, females and males used mainly the same foraging areas but exhibited spatial segregation afterwards (S2 Fig). In May, females showed a high-density location between 50°S to 48°S while males showed a high-density location between 54°S to 52°S. In June, females showed a high-density location between 42°S to 44°S while males foraged between 48°S to 46°S. In July and August, females were distributed throughout a larger foraging area (54°S to 42°S) than males (50°S to 46°S) and showed a high-density location between 48°S to 42°S, while males showed instead a high-density location between 48°S to 46°S. In September, males showed a high-density location between 50°S to 48°S while females showed a high-density location between 52°S to 50°S (S2 Fig). Finally, during the last month of the winter dispersal, females showed a high-density location in the south (between 56°S to54°S and 52°S and 50°S) while males showed a high-density location in the north (between 48°S to 46°S) (Fig 2).

Spatial analysis of bottom trawl fisheries showed that this fishery extended along all the Patagonian Shelf and presented a high-density fishing effort between 45°S to 40°S during all winter months. Also, in April there was a high-density effort between 50°S to 52°S and from May to September between 50°S to 45°S (S3A Fig). On the other hand, spatial analysis of shrimp fishery showed that this fishery occurred only between 50°S to 43°S and presented a high-density fishing effort around that area during all the months (S3B Fig).

**Fig 3:**
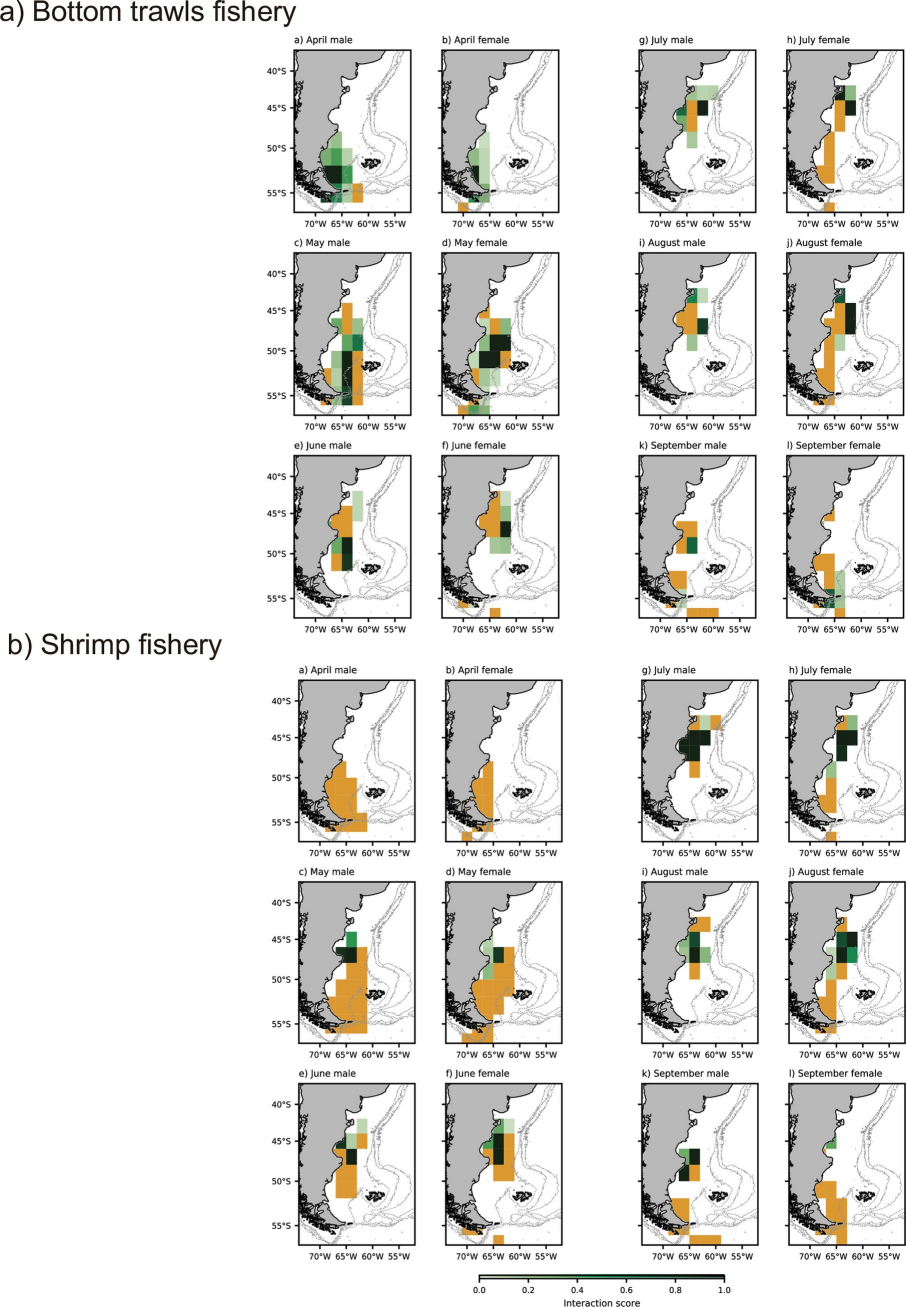
Overlap between Magellanic penguin locations and bottom trawl fishery (a) and shrimp fishery (b) along the Patagonia Shelf during their winter dispersal (April to September 2017), separated by sex and month. The orange grid represents penguins’ positions with 2° longitude x 2° latitude resolution without any overlap with fisheries positions. Light gray isobaths were obtained from [55, 56]. Fisheries data were obtained from MAGyP [43].

We found an overlap between bottom trawl fishing activity and Magellanic penguins’ locations during all months, in particular a high interaction score a) in April between 55°S to 53°Sfor both sexes; b) in May between 55°S to 50°S for males and between 53°S to 48°S for females; c) in June between 52°S to 48°S for males and between 48°S to 46°S for females; d) from July to August between 48°S to 43°S for both sexes; and e) in September between 50°S to 48°S for males and between 56°S to 53°S for females (Fig 3A).

We found an overlap between shrimp fishing activity and Magellanic penguins’ locations during May to September and no overlap during April for both sexes. In particular, we found a high interaction score a) from May to August between 50°S to 45°S for both sexes; and b) in September between 50°S to 46°S for males (Fig 3B).

We did not find differences between sexes in the overlap with bottom trawl (*X*^*2*^_*143*_ = 0.94, p = 0.33) or shrimp fisheries (*X*^*2*^_*143*_ = 0.21, p = 0.65). Also, we did not find differences between sexes with bottom trawl fisheries (*F*_*1*,*79*_ = 0.21, p = 0.65) and shrimp fishery interaction (*F*_*1*,*43*_ = 1.34, p = 0.25).

### Isotopic niche analysis

We did not find differences between sexes in δ^13^C (*F*_*1*, *22*_ *=* 4.1, p = 0.06) and δ^15^N values (*F*_*1*, *22*_ = 7.4, p = 0.3) (Table 2). Females and males overlapped their niches by 12%, which represented 16% of female´s niche and 48% of male´s niche (S4A Fig). However, we found that females showed larger isotopic niche widths (SEA_B_) relative to males (S4B Fig).

**Table 2:** Carbon (δ^13^C) and nitrogen (δ^15^N) stable isotope values of blood samples separated by sexes from Magellanic penguins. Values presented are mean ± SD. Different letters indicate significant differences within sex (p< 0.05).

Sex	n	δ^13^C (‰)		δ^15^N (‰)		C:N
		Mean ± SD Range		Mean ± SD Range		
Female	13	-18.3 ± 0.6 ^a^	-19.3 to -17.6	20.3 ± 1.1 ^b^	16.2 to 21.8	3.26
Male	11	-17.7 ± 0.5 ^a^	-18.7 to -17.2	21.0 ± 0.7 ^b^	19.8 to 21.9	3.25

### Trophic position

The estimated trophic position of Magellanic penguins differed between sexes (p = 0.02). Males showed higher values and a narrower distribution of trophic positions (Mean ± SD [C.I: 2.5% - 97.5%]: 5.4 ± 0.1 [5.1–5.6]) than females (Mean ± SD [C.I: 2.5% - 97.5%]: 4.8 ± 0.3 [4.3–5.3]).

## Discussion

We characterized the winter dispersal of Magellanic Penguins over the PS, its overlap with fisheries and the trophic niche of adult birds from a colony situated in the southern range of their breeding distribution. As most seabirds, Magellanic penguins expand their spatial range for foraging during the non-breeding season. Coherently, the tracked individuals used an extensive area of the PS as reported for individuals tagged in northern colonies [17, 18]. We detected spatial overlap with bottom trawl fishery during the whole range of their winter dispersal and with shrimp fishery from May to September. Although our sample size and spatial resolution are low, which is mainly due to the extreme conditions to which the geolocators are exposed and the challenging logistics involved in the collection of movement data over the winter period, we believe the results are conservative and confirm previous findings from this colony [19]. All individuals migrated from Martillo Island to the PS during winter and, except for one female, performed a long-range trip to the northern PS (~45°S). This supports the assumption that the destination areas recorded are representative of the areas exploited by the larger breeder’s population. On the other hand, despite a possible overestimation of overlap with fisheries by using a large pixel, regions of high overlap corresponded fairly well with regions of documented interaction with Magellanic penguins [22, 27, 57–59]. However, we acknowledge that inference drawn from these low sample sizes and spatial resolution deserves caution.

The winter dispersal depicted in this study differed from the dispersal from northern colonies. Cabo dos Bahías penguins (44° 56 ’S, 65° 33’W, [17]) used areas further north, while Quiroga Island penguins (47° 45 ’S, 65° 53’W, [18]) presented two strategies, some moving north and others moving southwards from their colony. In this sense, our results show an interesting staggering phenomenon, with Martillo penguins moving northwards into areas utilized by individuals from northerly colonies during the breeding period, which themselves moved further north for their winter dispersion. We also found that Magellanic penguins from Martillo Island and Cabo dos Bahías did not disperse as far away from their colony as individuals from Quiroga Island, which showed greater latitudinal ranges (Mean ± SD by colony: Quiroga Island: 2158.6 ± 257.8 km, Isla Martillo: 1362.8 ± 317.8 km, Cabo dos Bahías: 802.8 ± 304.3 km, 49, 50). Further tracking data studies from others Magellanic colonies are needed to understand colony-specific distributions during this period.

We expected to find differences between sexes in trip duration as male Magellanic penguins usually return earlier than females to claim a territory and to condition their nests [15]. However, we did not find significant differences in trip duration between sexes which could be due the fact that the sample size was limited and potentially too small to reach a strong conclusion.

We found latitudinal spatial segregation between sexes during May to August where females were located further north than males. In this sense, also during May to August, females Magellanic penguins from Cabo dos Bahías colony were mainly found further north than males [17]. In contrast, Magellanic penguins from Quiroga Island presented spatial sexual segregation but at a longitudinal level (east-west), where males were found farther from the coast than females [18]. Once again, we find similarities in the movement patterns between Cabo dos Bahías and Martillo Island and contrasting differences with Quiroga Island.

During the last month of the winter dispersal females were mainly located in southern Patagonian waters (between 50°S-55°S) while males preferred northern Patagonian waters (between 40°S-45°S) with little overlap between them. Also, females showed larger isotopic niche width and δ^13^C values range in comparison to males which could be related to this difference in the foraging areas or the result of a broader diet. However, in females an outlier with low δ^13^C and δ^15^N values could be influencing the calculated metrics (i.e. generating a wider SEA_C_). We have no GLS data for this individual, however, in light of the integrity of the sample, this individual could be feeding in a different foraging area or even on different prey with respect to the rest of females, which has a direct influence on it blood isotope values. Females’ showing a wider isotopic niche during the last month of the winter dispersal were also recorded at Quiroga Island colony [18]. In addition, the trophic position estimated for Martillo Island males’ showed high values during this period, which could be correlated to a diet composition dominated by high trophic position organisms and/or benthic prey, such as squid (e.g. *Illex argentines*, *Doryteuthis gahi*) and fish like common hake (*Merluccius hubbsi*) shorter than 30 cm of total length (e.g. [60, 61]). In contrast, females showed a lower trophic position in comparison to males, which could correlate to a diet dominated by lower trophic position and/or pelagic prey, such as fuegian sprat, shrimps and the pelagic form of squat lobster (*Munida gregaria*) [60, 62–64]. Also, females showed a greater variation in TP which could be associated, in part, with foraging in two areas with differing isotopic values at their base. A non-exclusive complimentary explanation of these results is related to males staying longer in the northern foraging grounds, where potential high nutrient food is available and perform a fast trip back to the colony. This would in turn explain their earlier arrival and at the same time the low variation in isotopic composition. In contrast, females return to the colony at a slower pace, which generates a more variable isotopic composition related to the latitudinal change in the baseline isotopic composition in the PS [65]. In this sense, the male’s higher body mass and size, and swimming capacity could allow for this behavior. For a better understanding of the trophic segregation between sexes, future studies could focus on the evaluation of the diet composition of each sex using mixing model analysis.

On the other hand, we found sexual segregation in their diving behavior with males using deeper waters than females throughout all the winter dispersal, as was found for the incubation period for the same colony [66] and during the winter dispersion for Cabo dos Bahías colony [17]. Taking into account that body size is allometrically correlated with diving capacities (larger-bodied individuals can dive deeper [67], such differences might be related to sexual size dimorphism since males’ Magellanic penguins are heavier [15] and larger than females [33] and thus can dive deeper. In any case, this behavior will help to reduce intraspecific competition in case of limited food availability when using similar areas during dispersal [66].

Based on sex-related differences found in Magellanic penguins spatial distribution and trophic position, a higher exposure to the potential fishing threat by either sex could generate a skewed sex ratio in the breeding population and thus could affect population viability [68]. Although females could be facing different exposure to by-catch than males given the dispersal into more northerly areas ([17] and this study) or closer to the coast [18], the higher female mortality found in wintering grounds off the Brazilian coast [23] could be skewed by the fact that females prefer those areas [17]. For our colony, however, we found no sex-specific susceptibility to fisheries during the winter period, despite of the spatial sexual segregation mentioned above and the spatially and temporarily clustered fishing effort pattern. In this sense, there appears to be no differential susceptibility to by-catch by sex. On the other hand, during the last month of the winter dispersal, the sexual differences found in the trophic positions could indicate different prey targets and this could be mirrored in their overlap with commercial fisheries (i.e., shrimp fisheries for females in contrast to bottom trawls fisheries for males).

We identified that the areas of high penguins-bottom trawl fishery overlap varied depending on the month of the Magellanic penguin’s winter dispersal. On the other hand, the area of high penguins-shrimp fishery overlap was focused between 50°S to 45°S during all the winter dispersal. In this sense, the penguins-shrimps fishery overlap seems to be more predictable through this period. However, the Argentine red shrimps’ stocks can show inter-annual variations which can result in changes in the fishing activity [69] (for example, changing the fishing effort towards others species). Also, [59] reported during the breeding season that the intensity of the spatial overlap of breeding Magellanic penguins with trawl fishing may vary between months and years, based on resource availability. Consequently, the intensity of penguin-trawl fisheries interaction seems to be spatiotemporal dependents throughout their annual cycle. This indicates that changes in fishing activity can modify the vulnerability of Magellanic penguin populations over short time periods which results in the need of a continuous monitoring to establish management and conservation strategies to protect them.

While we present an assessment of spatiotemporal overlap between fisheries and Magellanic penguins, we acknowledge that the relationship between overlap and interaction is complex. In this sense, our overlap score provides an estimation of potential relative risk. Despite these limitations, the timing and areas of overlap presented in this study will aid in targeting conservation measures to guarantee future Magellanic penguins’ yearly recruitment and long-term survival. It is important to highlight the importance of combining individual, fine-scale movement data with a large-scale spatiotemporal analysis framework for species risk assessment [70].

Several mitigation measures in order to reduce seabird-fisheries interaction are being used around the world, but with different degrees of implementation [44, 71–77]. In particular, Argentina created in 2010 the National Plan of Action‐Seabirds for the reduction of incidental mortality in all fisheries (Federal Fisheries Council, Resolution 03/10). In this sense, this large-scale fisheries analysis provides information that could support the protection of Magellanic penguins and the entire ecosystem.

## Conclusion

This study demonstrates that Magellanic penguins from Martillo Island showed sex-specific spatial-niche partitioning but this did not imply a sex-bias in the overlap with fisheries. We provide information on large scale information of penguin-fisheries overlap, which would help to identify important marine areas where the implementation of conservation plans and/or management guidelines are needed. Although we believe that knowledge of important at-sea areas are relevant for penguin conservation, a more holistic understanding of the penguin-fishery interactions will be necessary for the management and conservation of the marine environment as well as for global sustainability (i.e. by telecoupling analysis [78]).

## Supporting information

S1 Code Python script with spatial analysis of penguins-fisheries interaction. https://github.com/nlois/SpatialAnalysis_MagellanicPenguins.git
(TXT)

S1 Dataset Geolocators raw data set. https://doi.org/10.6084/m9.figshare.14388233.v3.
(TXT)

S1 Fig Individual migratory movements of Magellanic penguins during the winter dispersal (April-September 2017). Light gray isobaths were obtained from [55, 56].
(TIF)

S2 Fig Density plot distribution of daily positions obtained from Magellanic penguins separated by sex and month during their winter dispersal (April to September 2017, grid square: 2° latitude x 2° longitude). Light gray isobaths were obtained from [55, 56].
(TIF)

S3 Fig Density plot of fishing effort of bottom trawl fishery (a) and shrimp fishery (b) along the Patagonia Shelf during Magellanic penguins’ winter dispersal (April to September 2017, grid square: 2° latitude x 2° longitude). Score: 1: 0–60, 2: 60–200, 3: 200–800, 4: 800–6000, 5: 6000–10000. Light gray isobaths were obtained from [55, 56]. Fisheries data were obtained from MAGyP [43].
(TIF)

S4 Fig A) Standard ellipses corrected form small sample size (SEA_C_) estimated from δ^13^C and δ^15^N values from blood of females and males of Magellanic penguin collected after they return to Martillo Island (September 2017). Blood reflect the last month of their winter dispersal approximately (end August-beginning September 2017). B) Bayesian standard ellipse area (SEAB, presented in ‰^2^) from blood of females and males of Magellanic penguin. Black dots correspond to the mean SEAB for females and males, shaded boxes represent the 50%, 75%, and 95% credible intervals from dark to light gray.
(TIF)

S1 File GEBCO grid terms of use. (PDF)

## References

[1] Lehodey P, Alheit J, Barange M, Baumgartner T, Beaugrand G, Drinkwater K, et al. Climate Variability, Fish, and Fisheries. J Clim. 2006;19:5009–30.

[2] Boersma P, Rebstock G, Frere E, Moore S. Following the fish: Penguins and productivity in the South Atlantic. Ecol Monogr. 2009 Feb;79(1):59–76.

[3] Louge E, Reta R, Santos B, Hernandez D. Distribución de merluza (*Merluccius hubbsi* Marini, 1933) en el Mar Argentino (41°- 48°S) en relación con parámetros oceanográficos durante el invierno (1996–2003). Rev Biol Mar Oceanogr. 2009;44(2):497–510.

[4] Schreiber EA. Climate and weather effects on seabirds. In: Burger J, editor. Biology of Marine Birds. Boca Raton, FL: CRC Press; 2001. p. 179–207.

[5] González-Solís J, Croxall JP, Oro D, Ruiz X. Trans-equatorial migration and mixing inthe wintering areas of a pelagic seabird. Front Ecol Environ. 2007;5(6):297–301.

[6] Müller MS, Massa B, Phillips RA, Dell’omo G. Individual consistency and sex differences in migration strategies of scopoli’s shearwaters *Calonectris diomedea* despite year differences. Curr Zool. 2014;60(5):631–41.

[7] Bearhop S, Phillips RA, McGill R, Cherel Y, Dawson DA, Croxall JP. Stable isotopes indicate sex-specific and long-term individual foraging specialisation in diving seabirds. Mar Ecol Prog Ser. 2006 Apr 13;311:157–64.

[8] Phillips RA, McGill RAR, Dawson DA, Bearhop S. Sexual segregation in distribution, diet and trophic level of seabirds: Insights from stable isotope analysis. Mar Biol. 2011 Oct;158(10):2199–208.

[9] Barbraud C, Weimerskirch H. Climate and density shape population dynamics of a marine top predator. Proc Biol Sci. 2003;270:2111–211. doi: 10.1098/rspb.2003.2488 14561273 PMC1691492

[10] Raya Rey A, Trathan P, Pütz K, Schiavini A. Effect of oceanographic conditions on the winter movements of rockhopper penguins *Eudyptes chrysocome chrysocome* from Staten Island, Argentina. Mar Ecol Prog Ser. 2007;330:285–95.

[11] Thiebot JB, Cherel Y, Trathan PN, Bost CA. Inter-population segregation in the wintering areas of macaroni penguins. Mar Ecol Prog Ser. 2011;421:279–90.

[12] Frederiksen M, Moe B, Daunt F, Phillips RA, Barrett RT, Bogdanova MI, et al. Multicolony tracking reveals the winter distribution of a pelagic seabird on an ocean basin scale. Divers Distrib. 2012 Jun;18(6):530–42.

[13] McFarlane Tranquilla LA, Montevecchi WA, Hedd A, Fifield DA, Burke CM, Smith PA, et al. Multiple-colony winter habitat use by murres *Uria* spp. in the Northwest Atlantic Ocean: implications for marine risk assessment. Mar Ecol Prog Ser. 2013;472:287–303.

[14] Ouled-Cheikh J, Sanpera C, Becares J, Arcos JM, Carrasco JL, Ramirez F. Spatiotemporal analyses of tracking data reveal fine-scale, daily cycles in seabird-fisheries interactions. ICES J Mar Sci. 2020;77(7–8):2508–17.

[15] Boersma PD, Frere E, Kane O, Pozzi L, Pütz K, Raya Rey A, et al. Magellanic Penguin (*Spheniscus magellanicus*). In: Borboroglu PG, Boersma PD, editors. Penguin biology. Seattle, USA: University of Washington Press; 2013. p. 232–63.

[16] Parker G, Paterlini MC, Violante RA. El fondo marino. In: El Mar Argentino y sus recursos pesqueros. 1997. p. 65–87.

[17] Yamamoto T, Yoda K, Blanco GS, Quintana F. Female-biased stranding in Magellanic penguins. Curr Biol. 2019;29(1):12–3. doi: 10.1016/j.cub.2018.11.023 30620906

[18] Barrionuevo M, Ciancio J, Steinfurth A, Frere E. Geolocation and stable isotopes indicate habitat segregation between sexes in Magellanic penguins during the winter dispersion. J Avian Biol. 2019;1–12.

[19] Pütz K, Schiavini A, Rey AR, Lüthi BH. Winter migration of magellanic penguins (*Spheniscus magellanicus*) from the southernmost distributional range. Mar Biol. 2007;152(6):1227–35.

[20] Tamini LL, Perez JE, Chiaramonte GE, Cappozzo HL. Magellanic Penguin *Spheniscus magellanicus* and fish bycatch in the cornalito Sorgentinia incisa fishery at Puerto Quequén, Argentina. Atl Seabirds. 2002;4:109–14.

[21] Seco Pon JP, Copello S, Moretinni A, Lértora HP, Bruno I, Bastida J, et al. Seabird and marine-mammal attendance and by-catch in semi-industrial trawl fisheries in near-shore waters of northern Argentina. Mar Freshw Res. 2013;64(3):237–48.

[22] Paz JA, Seco Pon JP, Favero M, Blanco G, Copello S. Seabird interactions and by-catch in the anchovy pelagic trawl fishery operating in northern Argentina. Aquat Conserv Mar Freshw Ecosyst. 2018;28(4):850–60.

[23] Vanstreels RE, Adornes AC, Canabarro PL, Ruoppolo V, Amaku M, da Silva-Filho RP, et al. Female-biased mortality of Magellanic Penguins (*Spheniscus magellanicus*) on the wintering grounds. Emu. 2013;113(2):128–34.

[24] Fogliarini C, Bugoni L, Haimovici M, Resende Secchi E, Cardoso LG. High mortality of adult female Magellanic penguins by gillnet fisheries in southern Brazil. Aquat Conserv Mar Freshw Ecosyst. 2019;29(10):1657–64.

[25] Gownaris NJ, Boersma PD. Sex-biased survival contributes to population decline in a long-lived seabird, the Magellanic Penguin. Ecol Appl. 2019;29(1). 10.1002/eap.1826 PMC6849821 30601594

[26] Ainley DG. Feeding methods in seabirds: a comparison of polar and tropical nesting communities in the eastern Pacific Ocean. In: Llano GA, editor. Adaptations within Antarctic ecosystems. Washington DC: Smithsonian Institution Scholarly Press; 1997. p. 669–85.

[27] Gandini PA, Frere E, Pettovello AD, Cedrola P V. Interaction between Magellanic penguins and shrimp fisheries in Patagonia, Argentina. Condor. 1999;101(4):783–9.

[28] Pütz K, Ingham RJ, Smith JG, Croxall JP. Population trends, breeding success and diet composition of gentoo *Pygoscelis papua*, magellanic *Spheniscus magellanicus* and rockhopper *Eudyptes chrysocome* penguins in the Falkland Islands. Polar Biol. 2001;24:793–807.

[29] Diez MJ, Cabreira AG, Madirolas A, De Nascimento JM, Scioscia G, Schiavini A, et al. Winter is cool: spatio-temporal patterns of the squat lobster *Munida gregaria* and the Fuegian sprat Sprattus fuegensis in a sub-Antarctic estuarine environment. Polar Biol. 2018;41(12):2591–605.

[30] Giller P. Community Structure and the Niche. Dordrecht: Springer Netherlands; 1984.

[31] Villasante S, Macho G, De Rivero JI, Divovich E, Zylich K, Harper S, et al. Reconstruction of marine fisheries catches in Argentina (1950–2010). 2015. Available from: www.fishbase.org

[32] Allega L, Braverman M, Cabreira AG, Campodónico S, Colonello JH, Derisio C, et al. Estado del conocimiento biológico pesquero de los principales recursos vivos y su ambiente, con relación a la exploración hidrocarburífera en la Zona Económica Exclusiva Argentina y adyacencias. INIDEP. Boschi EE, Aristizabal Abud EO, Ivanovic ML, editors. Mar del Plata; 2019. 123 p.

[33] Gandini PA, Frere E, Holik TM. Implicancias de las diferencias en el tamaño corporal entre colonias para el uso de medidas morfométricas como método de sexado en *Spheniscus magellanicus*. El Hornero. 1992;13:211–3.

[34] Hobson KA, Gloutney ML, Gibbs HL. Preservation of blood and tissue samples for stable-carbon and stable-nitrogen isotope analysis. Can J Zool. 1997;75:720–1723.

[35] R Core Team. R: A language and evironment for statistical computing, version 4.1.0. Viena, Austria: R Foundation for Statistical Computing; 2021. Available from: http://www.r-project.org/

[36] Ekstrom P. Error measures for template-fit geolocation based on light. Deep Sea Res Part II Top Stud Oceanogr. 2007;54:392–403.

[37] Wilson R, Ducamp JJ, Rees WG, Culik BM, Niekamp K. Estimation of location: Global coverage using light intensity. In: Priede IG, Swift SM, editors. Wildlife Telemetry remote monitoring and tracking of animals. England: Ellis Horwood Series in Environmental Management, Science and Technology; 1992. p. 131–4.

[38] Merkel B, Phillips RA, Descamps S, Yoccoz NG, Moe B, Strøm H. A probabilistic algorithm to process geolocation data. Mov Ecol. 2016;4–26. doi: 10.1186/s40462-016-0069-6 27891228 PMC5116194

[39] Stokes DL, Boersma PD. Satellite tracking of Magellanic penguin migration. Condor. 1998;100:376–81.

[40] Teo SLH, Boustany A, Blackwell S, Walli A, Weng KC, Block BA. Validation of geolocation estimates based on light level and sea surface temperature from electronic tags. Mar Ecol Prog Ser. 2004;283:81–98.

[41] Shaffer SA, Tremblay Y, Awkerman JA, Henry RW, Teo SLH, Anderson DJ, et al. Comparison of light- and SST-based geolocation with satellite telemetry in free-ranging albatrosses. Mar Biol. 2005;147:833–43.

[42] Pinheiro J, Bates DM, Saikat D, Sarkar D, Team RC. Linear and Nonlinear Mixed Effects Models: 3.2.5. R package version 3.1–131. 2015.

[43] Martinez Puljak G, Navarro G, Prosdocimi L, Sanchez R, Lenicov Remes M. Mejora de la resolución espacial de la información estadística de la flota pesquera Argentina. 2018.

[44] González-Zevallos D, Yorio P, Caille G. Seabird mortality at trawler warp cables and a proposed mitigation measure: A case of study in Golfo San Jorge, Patagonia, Argentina. Biol Conserv. 2007;136:108–16.

[45] Yorio P, Quintana F, Dell’arciprete P, González-Zevallos D. Spatial overlap between foraging seabirds and trawl fisheries: Implications for the effectiveness of a marine protected area at Golfo San Jorge, Argentina. Bird Conserv Int. 2010;20:320–34.

[46] Dupuis B, Amélineau F, Tarroux A, Bjørnstad O, Bråthen S, Danielsen J, et al. Light-level geolocators reveal spatial variations in interactions between northern fulmars and fisheries. Mar Ecol Prog Ser. 2021; Available from: 10.3354/meps13673

[47] Barquete V, Strauss V, Ryan PG. Stable isotope turnover in blood and claws: A case study in captive African Penguins. J Exp Mar Bio Ecol. 2013;448:121–7.

[48] Jackson AL, Inger R, Parnell AC, Bearhop S. Comparing isotopic niche widths among and within communities: SIBER—Stable Isotope Bayesian Ellipses in R. J Anim Ecol. 2011;80(3):595–602. doi: 10.1111/j.1365-2656.2011.01806.x 21401589

[49] Quezada-Romegialli C, Jackson AL, Hayden B, Kahilainen KK, Lopes C, Harrod C. tRophicPosition, an r package for the Bayesian estimation of trophic position from consumer stable isotope ratios. Methods Ecol Evol. 2018;9(6):1592–9.

[50] Saporiti F, Bearhop S, Vales DG, Silva L, Zenteno L, Tavares M, et al. Latitudinal changes in the structure of marine food webs in the Southwestern Atlantic Ocean. Mar Ecol Prog Ser. 2015;538:23–34.

[51] Balza U, Lois NA, Polito MJ, Pütz K, Salom A, Raya Rey A. The dynamic trophic niche of an island bird of prey. Ecol Evol. 2020;00:1–13. doi: 10.1002/ece3.6856 33209286 PMC7663050

[52] Francey RJ, Allisoni CE, Etheridgei DM, Trudingeri CM. A 1000-year high precision record of δ^13^C in atmospheric CO. Tellus. 1999;51:170–93.

[53] Indermühle A, Stocker TF, Joos F, Fischer H, Smith HJ, Wahlen M, et al. Holocene carbon-cycle dynamics based on CO2 trapped in ice at Taylor Dome, Antarctica. Nature. 1999;398:121−126. Available from: www.nature.com

[54] Ciancio JE, Righi C, Faiella A, Frere E. Blood-specific isotopic discrimination factors in the Magellanic penguin (*Spheniscus magellanicus*). Rapid Commun Mass Spectrom. 2016 Aug 30;30(16):1865–9. doi: 10.1002/rcm.7661 27476661

[55] NOAA National Geophysical Data Center. ETOPO1 1 Arc-Minute Global Relief Model. NOAA National Centers for Environmental Information. Accessed [date]; 2009.

[56] Amante C, Eakins B. ETOPO1 1 Arc-Minute Global Relief Model: Procedures, Data Sources and Analysis. NOAA Technical Memorandum NESDIS NGDC-24. National Geophysical Data Center. NOAA National Centers for Environmental Information. doi: 10.7289/V5C8276M [access date]; 2009.

[57] González-Zevallos D, Yorio P. Seabird use of discards and incidental captures at the Argentine hake trawl fishery in the Golfo San Jorge, Argentina. Mar Ecol Prog Ser. 2006;316:175–83.

[58] González-Zevallos D, Yorio P, Svagelj WS. Seabird attendance and incidental mortality at shrimp fisheries in Golfo San Jorge, Argentina. Mar Ecol Prog Ser. 2011;432:125–35.

[59] Yorio P, Suárez N, Dell’Arciprete P, Marinao C, Góngora M, Pichegru L, et al. Spatial use of multiple jurisdictions by Magellanic penguins and assessment of potential conflicts in the face of changing trawl fisheries scenarios. Mar Ecol Prog Ser. 2021;(658):219–36.

[60] Ciancio JE, Pascual MA, Botto F, Frere E, Iribarne O. Trophic relationships of exotic anadromous salmonids in the southern Patagonian Shelf as inferred from stable isotopes. Limnol Oceanogr. 2008;53(2):788–98.

[61] Silva L, Saporit F, Vales D, Tavares M, Gandini P, Crespo EA, et al. Differences in diet composition and foraging patterns between sexes of the Magellanic penguin (*Spheniscus magellanicus*) during the non-breeding period as revealed by δ^13^C and δ^15^N values in feathers and bone. Mar Biol. 2014;161(5):1195–206.

[62] Forero MG, Bortolotti GR, Hobson KA, Donazar JA, Bertelloti M, Blanco G. High trophic overlap within the seabird community of Argentinean Patagonia: a multiscale approach. J Anim Ecol. 2004;73:789–801.

[63] Riccialdelli L, Becker YA, Fioramonti NE, Torres M, Bruno DO, Raya Rey A, et al. Trophic structure of southern marine ecosystems: a comparative isotopic analysis from the Beagle Channel to the oceanic Burdwood Bank area under a wasp-waist assumption. Mar Ecol Prog Ser. 2020;655: 1–27.

[64] Dodino S, Riccialdelli L, Polito M, Pütz K, Raya Rey A. Inter-annual variation in the trophic niche of Magellanic penguins *Spheniscus magellanicus* during the pre-molt period in the Beagle Channel. Mar Ecol Prog Ser. 2020;655:215–25.

[65] Lara RJ, Alder V, Franzosi CA, Kattner G. Characteristics of suspended particulate organic matter in the southwestern Atlantic: Influence of temperature, nutrient and phytoplankton features on the stable isotope signature. J Mar Syst. 2010;79(1–2):199–209.

[66] Raya Rey A, Pütz K, Scioscia G, Lüthi B. Sexual differences in the foraging behaviour of Magellanic Penguins related to stage of breeding. Emu. 2012;112:90–6.

[67] Noren SR, Williams TM, Pabst DA, McLellan WA, Dearolf JL. The development of diving in marine endotherms: Preparing the skeletal muscles of dolphins, penguins, and seals for activity during submergence. J Comp Physiol Biochem. 2001;171:127–34. 10.1007/s003600000161 11302529

[68] Wearmouth VJ, Sims DW. Sexual segregation in marine fish, reptiles, birds and mammals: behaviour patterns, mechanisms and conservation implications. In: Advances in Marine Biology. 2008. p. 107–70. doi: 10.1016/S0065-2881(08)00002-3 18929064

[69] Góngora ME, González-Zevallos D, Pettovello A, Mendía L. Caracterización de las principales pesquerías del golfo San Jorge Patagonia, Argentina. Vol. 40, Latin American Journal of Aquatic Research. 2012. p. 1–11.

[70] Croxall J, Small C, Sullivan B, Wanless R, Frere E, Lascelles B, et al. Appropriate scales and data to manage seabird-fishery interactions: Comment on Torres et al. (2013). Mar Ecol Prog Ser. 2013;493:297–300.

[71] Abraham ER, Pierre JP, Middleton DAJ, Cleal J, Walker NA, Waugh SM. Effectiveness of fish waste management strategies in reducing seabird attendance at a trawl vessel. Fish Res. 2009;(95):210–219.

[72] Bull LS. New mitigation measures reducing seabird by-catch in trawl fisheries. Fish Fish. 2009 Dec;10(4):408–27.

[73] Melvin EF, Dietrich KS, Fitzgerald S, Cardoso T. Reducing seabird strikes with trawl cables in the Pollock catcher‐processor fleet in the eastern Bering Sea. Polar Biol. 2011;(34):215–226.

[74] Pierre JP, Abraham E, Middleton DAJ, Cleal J, Bird R, Walker N, et al. Reducing interactions between seabirds and trawl fisheries: Responses to foraging patches provided by fish waste batches. Biol Conserv. 2010;(143):2779–2788.

[75] Sullivan BJ, Brickle P, Reid TA, Bone DG, Middleton DAJ. Mitigation of seabird mortality on factory trawlers: Trials of three devices to reduce warp cable strikes. Polar Biol. 2006 Aug;29(9):745–53.

[76] Tamini LL, Chavez LN, Góngora ME, Yates O, Rabuffetti FL, Sullivan B. Estimating mortality of black-browed albatross (*Thalassarche melanophris*, Temminck, 1828) and other seabirds in the Argentinean factory trawl fleet and the use of bird-scaring lines as a mitigation measure. Polar Biol. 2015;(38):1867–1879.

[77] Tamini LL, Chavez LN, Dellacasa RF, Crawford R, Frere E. Incidental capture of seabirds in Argentinean side-haul trawlers. Bird Conserv Int. 2020: 1–14.

[78] Raya Rey A, Huettmann F. Telecoupling analysis of the Patagonian Shelf: A new approach to study global seabird-fisheries interactions to achieve sustainability. J Nat Conserv. 2020;53(125748).

