# Supplementary figures and images for: Sex-specific spatial use of the winter foraging areas by Magellanic penguins and assessment of potential conflicts with fisheries during winter dispersal
Source: PLoS One. 2021 Aug 20;16(8):e0256339. doi: 10.1371/journal.pone.0256339 (PMC8378684; doi:10.1371/journal.pone.0256339)

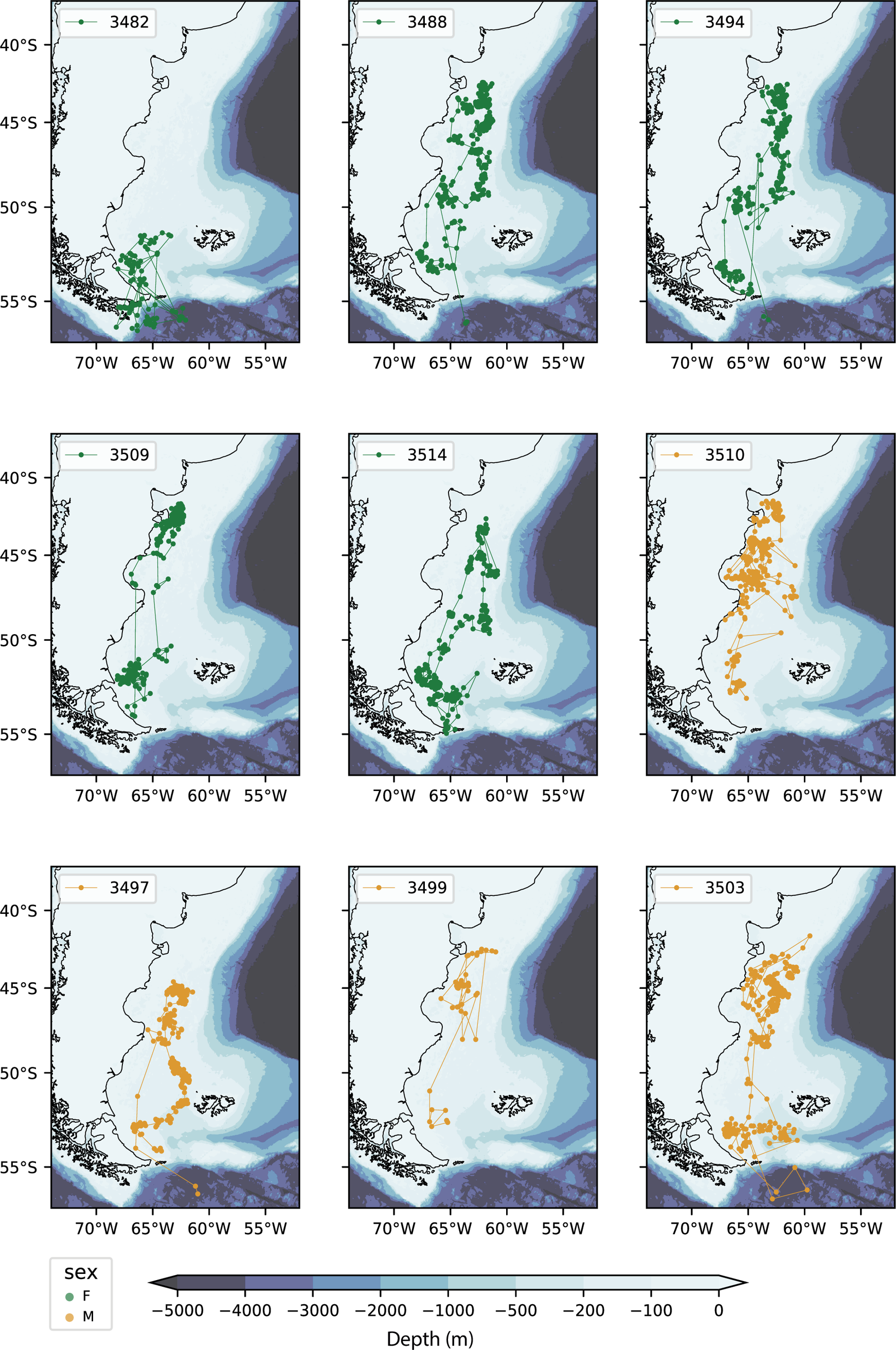

Supplement: S1 Fig — Light gray isobaths were obtained from [55, 56]. (TIF) [file pone.0256339.s003.tif]

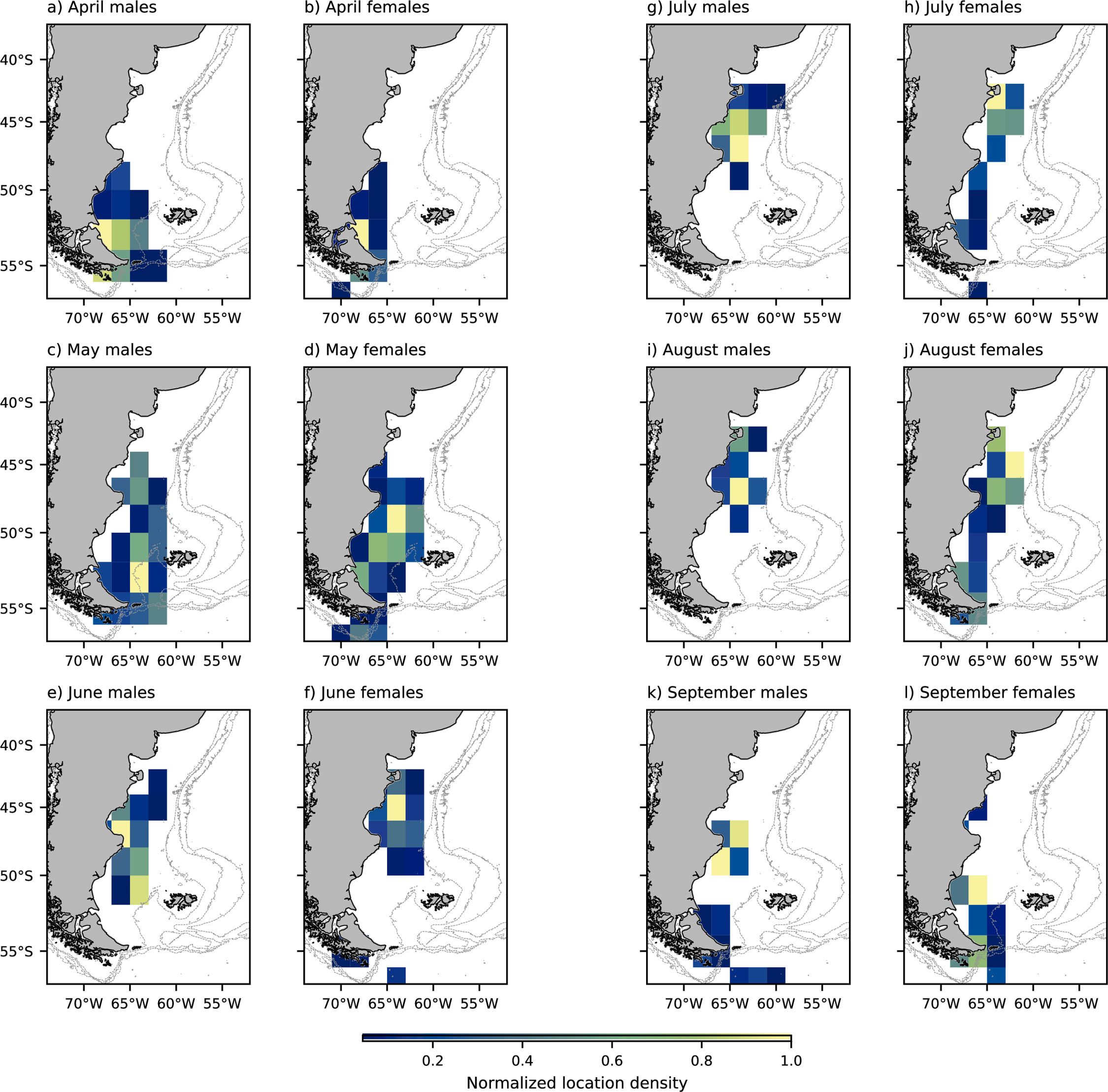

Supplement: S2 Fig — Light gray isobaths were obtained from [55, 56]. (TIF) [file pone.0256339.s004.tif]

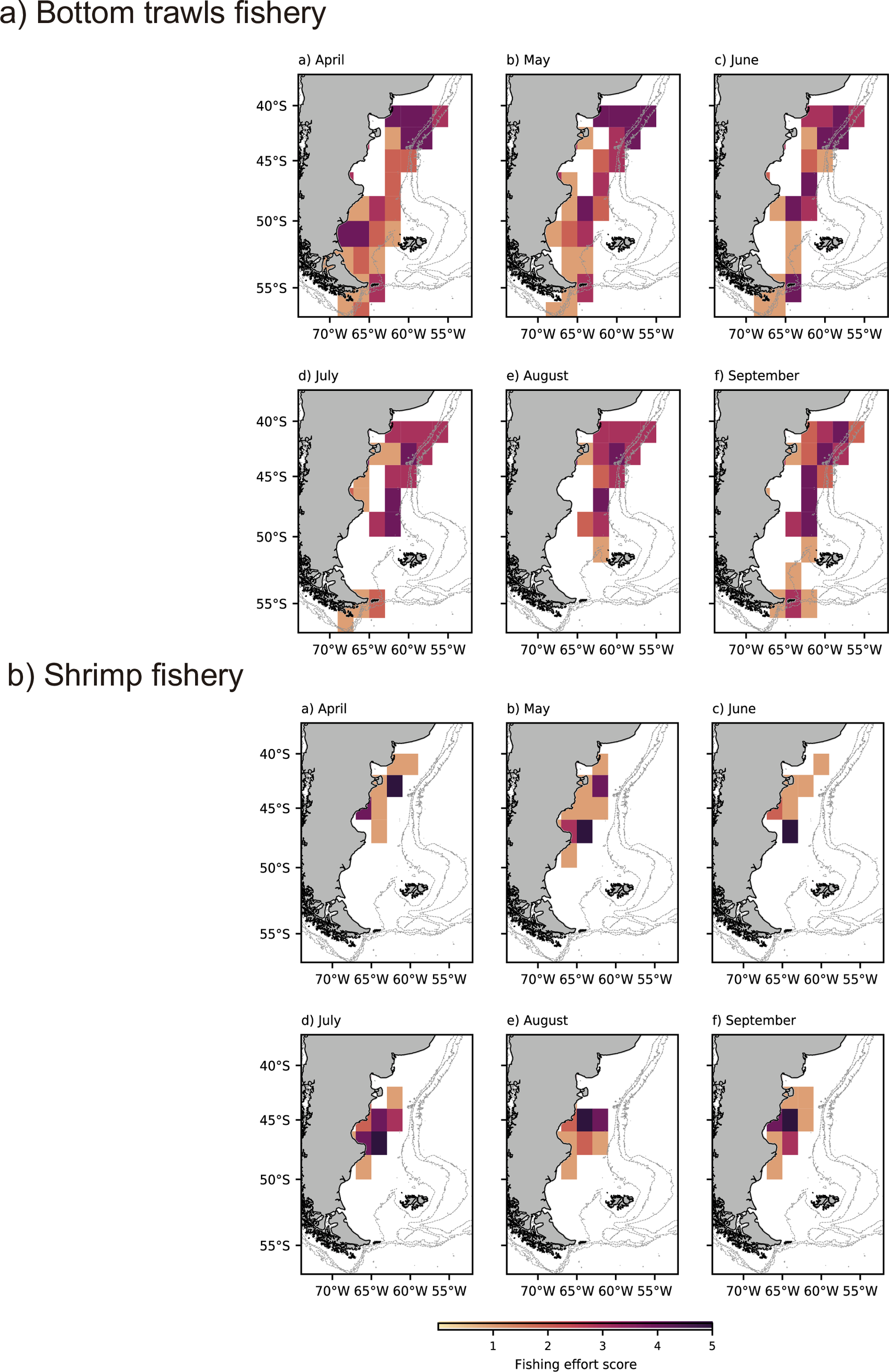

Supplement: S3 Fig — Density plot of fishing effort of bottom trawl fishery (a) and shrimp fishery (b) along the Patagonia Shelf during Magellanic penguins’ winter dispersal (April to September 2017, grid square: 2° latitude x 2° longitude). Score: 1: 0–60, 2: 60–200, 3: 200–800, 4: 800–6000, 5: 6000–10000. Light gray isobaths were obtained from [55, 56]. Fisheries data were obtained from MAGyP [43]. (TIF) [file pone.0256339.s005.tif]

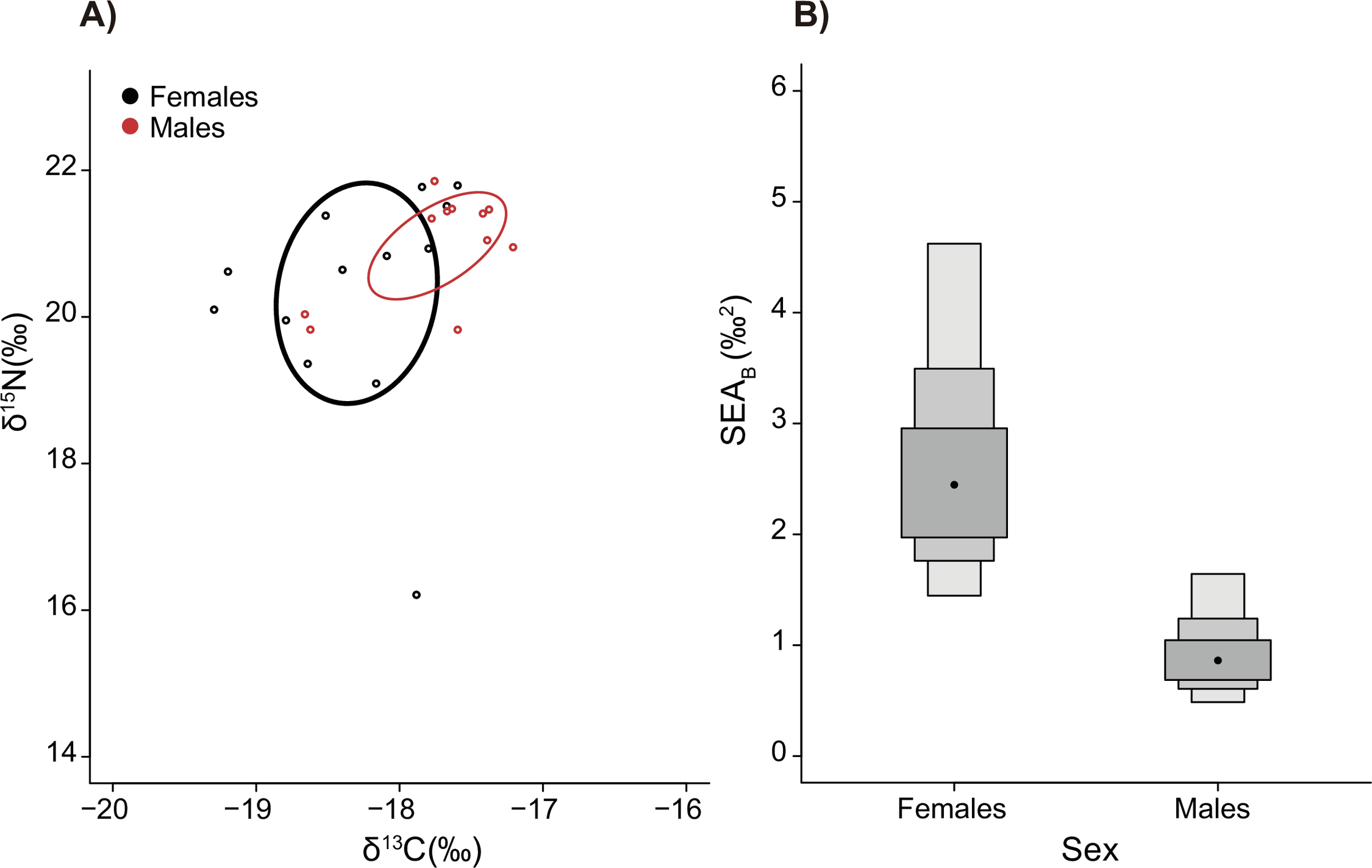

Supplement: S4 Fig — A) Standard ellipses corrected form small sample size (SEAC) estimated from δ13C and δ15N values from blood of females and males of Magellanic penguin collected after they return to Martillo Island (September 2017). Blood reflect the last month of their winter dispersal approximately (end August-beginning September 2017). B) Bayesian standard ellipse area (SEAB, presented in ‰2) from blood of females and males of Magellanic penguin. Black dots correspond to the mean SEAB for females and males, shaded boxes represent the 50%, 75%, and 95% credible intervals from dark to light gray. (TIF) [file pone.0256339.s006.tif]
