# Supplementary material for: Sex-specific spatial use of the winter foraging areas by Magellanic penguins and assessment of potential conflicts with fisheries during winter dispersal
Source: PLoS One. 2021 Aug 20;16(8):e0256339. doi: 10.1371/journal.pone.0256339 (PMC8378684; doi:10.1371/journal.pone.0256339)
Supplement: S1 File — (PDF) [file pone.0256339.s007.pdf]

Terms of Use of The GEBCO Grid and derived information products

Scope

- These terms of use apply to The GEBCO Grid and other GEBCO-derived information products
- For brevity 'The GEBCO Grid' is used throughout and should be interpreted as meaning The GEBCO Grid and other GEBCO-derived information products
- Bathymetric Data refers to measurements made by various instruments of the ocean depth, associated ocean properties and the supporting metadata
- Information products are the result of applying algorithms, mathematical techniques, scientific theory and Intellectual Property to data to create useful, derived values.
- As the GEBCO Grid is created by interpolating, applying algorithms and mathematical techniques to bathymetric data, GEBCO considers the GEBCO Grid to be an information product
- GEBCO does not provide the underlying source bathymetric data when distributing the GEBCO Grid

Terms of use

The GEBCO Grid is placed in the public domain and may be used free of charge.

Use of the GEBCO Grid indicates that the user accepts the conditions of use and disclaimer information given below.

Users are free to:

- Copy, publish, distribute and transmit The GEBCO Grid
- Adapt The GEBCO Grid
- Commercially exploit The GEBCO Grid, by, for example, combining it with other information, or by including it in their own product or application

Users must:

- Acknowledge the source of The GEBCO Grid. A suitable form of attribution is given in the documentation that accompanies The GEBCO Grid.
- Not use The GEBCO Grid in a way that suggests any official status or that GEBCO, or the IHO or IOC, endorses any particular application of The GEBCO Grid.
- Not mislead others or misrepresent The GEBCO Grid or its source.

Disclaimer

- The GEBCO Grid should NOT be used for navigation or for any other purpose involving safety at sea.

- The GEBCO Grid is made available 'as is'. While every effort has been made to ensure reliability within the limits of present knowledge, the accuracy and completeness of The GEBCO Grid cannot be guaranteed. No responsibility can be accepted by GEBCO, IHO, IOC, or those involved in its creation or publication for any consequential loss, injury or damage arising from its use or for determining the fitness of The GEBCO Grid for any particular use.
- The GEBCO Grid is based on bathymetric data from many different sources of varying quality and coverage.
- As The GEBCO Grid is an information product created by interpolation of measured data, the resolution of The GEBCO Grid may be significantly different to that of the resolution of the underlying measured data.

Data set attribution

If the data sets are used in a presentation or publication then we ask that you acknowledge the source. This should be of the form:

GEBCO Compilation Group (2020) GEBCO 2020 Grid (doi:10.5285/a29c5465-b138-234d-e053-6c86abc040b9).
